# Pathophysiology of Hyponatremia in Children

**DOI:** 10.3389/fped.2017.00213

**Published:** 2017-10-16

**Authors:** Jakub Zieg

**Affiliations:** ^1^Second Faculty of Medicine, Department of Pediatrics, Motol University Hospital, Charles University in Prague, Praha, Czechia

**Keywords:** hyponatremia, pathophysiology, children, antidiuretic hormone, baroreceptor, osmoreceptor

## Abstract

Hyponatremia is a common electrolyte disorder in children. It is generally defined as plasma sodium of less than 135 mmol/l. Sodium homeostasis is essential for maintaining intravascular volume and is tightly linked to water balance. Plasma water volume is regulated mainly by the secretion of an antidiuretic hormone (ADH) and by the thirst mechanism. ADH is synthesized in the hypothalamus and stored in the posterior hypophysis. It binds to V2 receptors in the distal nephron and induces translocation of aquaporin water channels in the plasma membrane to retain water. There are two main types of receptors involved in the control of the body water balance—osmoreceptors and baroreceptors. Osmoreceptors reside in hypothalamus and respond to changes of extracellular fluid (ECF) osmolality. Baroreceptors are mechanoreceptors that sense blood pressure in the vessel wall. Response reflexes from baroreceptors influence sympathetic outflow, vessel tonus, and cardiac output. An increase of 1% of plasma osmolality may cause an increase in ADH levels, while the threshold of volume receptors for ADH secretion is higher. However, significant hypotension is a more potent stimulus for ADH secretion than increased osmolality. The main cause of pediatric hyponatremia is an abundance of free water. This may occur in hypovolemic children with low ECF volume, normovolemic patients with inappropriately increased ADH secretion, and also in hypervolemic individuals with decreased effective circulating volume and appropriately increased ADH levels. Proper understanding of the pathophysiology of hyponatremic states is essential for establishing the correct diagnosis and appropriate therapy.

## Introduction

Sodium is the major cation in extracellular fluid (ECF). Water shifts often result in changes of sodium plasma concentration, thus dysnatremias commonly reflect water balance disorders. Total body water (TBW) is the total amount of fluid in the body and makes up a significant fraction of the human body. Premature infants have the highest TBW content of 75–80%. TBW decreases with age—70% in term newborns and 60% by age of 1 year. TBW is distributed in two major departments, about two-thirds of TBW is within cells—intracellular fluid; the remaining one-third—extracellular volume—is outside cells and is further divided in the smaller compartments: plasma, interstitial fluid, and transcellular fluid (1). Hyponatremia, defined as a serum sodium <135 mmol/l, is the most common electrolyte disorder encountered in clinical practice (2, 3). It is often the consequence of free water excess due to appropriate or inappropriate antidiuretic hormone (ADH) secretion, and sodium wasting is much less prevalent (3). Hyponatremia can occur with different volume status depending on the underlying disorder. The aim of this article is to review the pathophysiological principles of hyponatremia in various clinical situations.

## Sodium and Water Balance

Sodium serum concentration is kept within the normal range by several mechanisms. Ion homeostasis and water balance are controlled by complex neurohumoral regulations. The autonomic nervous system plays an important role in renal hemodynamics, renin secretion, and tubular fluid reabsorption. Functional and animal studies support the clear link between sympathetic nerve stimulation and both renin release (4, 5) and proximal tubular fluid reabsorption (6). While short-term fluid regulation in response to changes in sodium and water balance is performed by direct actions of the autonomic nervous system, hormones—ADH and aldosterone—are responsible for long-term effects on sodium and water reabsorption (7). ADH, a neurohypophyseal hormone, plays a key role in water regulation. It is produced in a specific population of neurons in the supraoptic and paraventricular nucleus of the hypothalamus. Secretory granules containing ADH move down the axons to the neurohypophysis, where they are stored and secreted in response to increased serum osmolality or non-osmotic factors. A small (1%) increase of osmolality and a high (5–10%) decrease in blood volume are effective stimuli both for ADH secretion and thirst (8).

Hypovolemia is a less sensitive stimulus for ADH release; however, the body prioritizes volume over osmolality when there is a significant decrease in intravascular fluid volume, which may counteract the effect of hypoosmolality and enhance ADH secretion. The kidney does not sense serum sodium; it regulates sodium reabsorption to maintain volume homeostasis, not to regulate sodium balance. Thus, sodium is reabsorbed to preserve intravascular volume in response to renal perfusion (3). Osmoreceptors, sensory cells, which detect changes in serum osmolality, reside in the supraoptic, paraventricular nuclei of the hypothalamus and in the organum vasculosum of the lamina terminalis (9). Hypoosmolality leads to increased osmoreceptor volume due to water movement through the channels into the cells and subsequent accelerated action potential discharge resulting in decreased secretion of ADH into the blood stream. Conversely, hyperosmolal environment causes cell contraction, deceleration of action potential and rise in ADH release into the circulation. ADH acts on specialized receptors (V2) on the basolateral membrane of renal collecting ducts. Activation of V2 receptors stimulates an intracellular cascade—activation of adenylate cyclase, cyclic adenosine monophosphate, and protein kinase A (PKA) formation resulting in insertion of aquaporin (AQP2) channels into the luminal membrane. PKA independent pathways have also been implicated in the regulation of ADH-induced AQP2 expression (10). Water then moves through these channels into the cell and gets to the blood stream through AQP3 and AQP4 water channels in the basolateral membrane (11). Vasopressin also increases the number of epithelial sodium receptors (ENaC) causing enhanced sodium reabsorption and contributing to water retention (12). Osmotic and non-osmotic stimuli lead also to an increase in thirst, sensed peripherally as the sensation of a dry mouth. Interestingly, thirst develops later with 5–10 mmol/kg higher serum osmolality compared with ADH secretion (13). This centrally regulated mechanism prevents hypernatremia in normal subjects as long as they have access to water. Satisfying thirst by the contact of lips and mouth with water shows that control of thirst is more complicated. Not only does information about plasma osmolality and volume enter the lamina terminalis in the hypothalamus but also these neurons are directly modulated by signals from the oral cavity during drinking and eating. However, it is still unknown how the peripheral sensations in the oral cavity are detected (14).

## Baroreceptors

Effective intravascular volume is sensed by baroreceptors, which are sensors in the vascular system. High pressure receptors in the aorta and carotic sinus detect mean arterial pressure, and afferent parasympathetic fibers transmit this signal to the vasomotor center regulating the response to maintain arterial pressure in the normal range. Arterial underfilling leads to ADH secretion, activation of renin–angiotensin–aldosterone system (RAAS), vasoconstriction, and an increased heart rate. Production of natriuretic peptides (NP) is inhibited at the same time. These changes enhance sodium reabsorption and water retention (15). Decreased effective intravascular volume may be caused by decreased cardiac output in patients with heart failure or decreased intrathoracic volume. Other causes of arterial underfilling include sepsis and cirrhosis, which may be associated with arterial vasodilation. Effective intravascular volume contraction is associated with volume depletion, and subsequent free water intake is a frequent cause of hyponatremia. Low pressure receptors sense mechanical stretch in atria and great veins. Increased pressure in these low pressure compartments causes sympathetic nerve-dependent natriuresis and diuresis (16). High pressure receptors are more important in regulating ADH secretion and sympathetic tone compared with low pressure receptors. This is apparent, for example, in patients with congestive heart failure with increased volume and pressure in heart cavities, where low pressure receptors would be expected to lower sympathetic tone and ADH secretion in these individuals (17).

As noted, our body is not able to sense sodium; however, there are specialized receptors that are able to detect tubular flow and its sodium concentration. They are a part of the juxtaglomerular apparatus, a specialized structure of the nephron where the afferent arteriole and distal convoluted tubule come in contact. It contains three types of cells:

1. juxtaglomerular cells, modified smooth muscle cells of the afferent arteriole, which secrete renin;
2. macula densa cells of the distal convoluted tubule, which sense tubular filtrate flow and sodium concentration;
3. juxtaglomerular/extraglomerular mesangial cells (Lacis cells) located between the afferent and efferent arteriole. Their function is not clear.

Increased tubular filtrate flow and sodium concentration are sensed by macula densa cells. This information is sent to juxtaglomerular cells located in the walls of arterioles to decrease the glomerular filtration rate (GFR) by a tubuloglomerular feedback mechanism. By contrast, decreased tubular flow results in increased GFR. The effect on renal arterioles is essential for GFR regulation. This mechanism prevents the loss of body fluids in states of glomerular hyperfusion (13, 18).

Also, RAAS is important in intravascular volume maintenance, blood pressure, and electrolyte balance regulation. Four main signals are responsible for the release of renin from juxtaglomerular cells (Table 1) (13, 19).

**Table 1:** Major triggers of renin release.

Reduction of renal perfusion pressure
Sympathetic nerve activation
Decreased sodium delivery to the distal tubule
Reduced levels of locally acting hormones (angiotensin II and atrial natriuretic peptide)

Renin catalyzes the conversion of angiotensinogen, protein synthesized in the liver, into angiotensin I. The angiotensin-converting enzyme further converts, mainly within the lungs, angiotensin I to angiotensin II, which has several important functions to maintain the blood pressure and intravascular volume (Table 2) (13, 20).

**Table 2:** Actions of angiotensin II in blood pressure and intravascular volume regulation.

Vasoconstriction resulting to increased blood pressure
Sodium and water retention
Release of aldosterone with sodium and water preserving effect
Stimulation of antidiuretic hormone release
Stimulation of thirst
Activation of noradrenaline release from sympathetic nerve endings
Stimulation of vascular and cardiac hypertrophy

**Figure 1:**
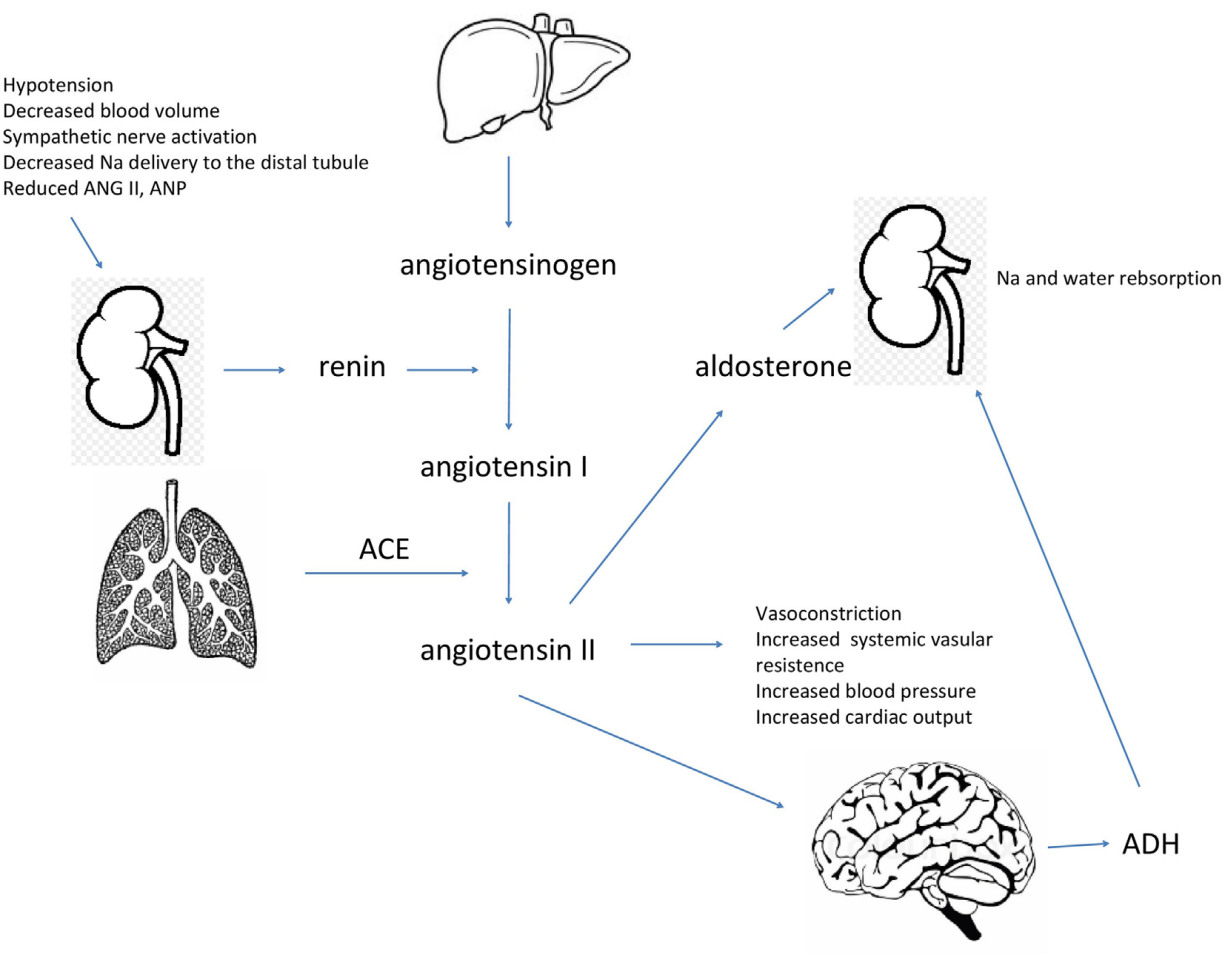
Specific triggers are necessary for the secretion of renin into bloodstream by the kidneys. Renin hydrolyzes angiotensinogen to produce angiotensin I, which is further converted to angiotensin II by the angiotensin-converting enzyme. Angiotensin II acts to increase blood volume and maintain blood pressure, it also stimulates the release of aldosterone and antidiuretic hormone (ADH) to retain water and sodium.

The RAAS system is essential for body fluid retention and blood pressure maintenance. There are also peptide hormones, NP, which have the opposite effect. Atrial natriuretic peptide and brain natriuretic are hormones secreted from the heart. Their biologic actions are vasodilation, stimulation of diuresis, natriuresis, and to decrease arterial blood pressure and systemic vascular resistance. They also suppress aldosterone and ADH release. Sodium excretion is enhanced both by an increase in GFR and suppression of salt reabsorption. NP are secreted in response to perceived increase of body fluid volume sensed as a wall stretch of the heart cavities. Sympathetic and angiotensin II stimulation also enhance secretion of NP, which are distributed to target organs *via* the circulation. NP receptors have been identified in blood vessels, adrenal glands, and kidneys. Activation of these receptors leads to the synthesis of cyclic guanosine monophosphate, which mediates the action of these peptides (13, 20, 21). Figure 1 shows the regulation of sodium and water balance in the body.

The etiology of hyponatremia is highly variable, and correct diagnosis is based on proper understanding of the pathophysiological mechanisms of this electrolyte disorder. Detailed history, physical examination, and basic laboratory tests are necessary for the evaluation of hyponatremic states. Initially, it is important to assess serum osmolality and tonicity. Osmolality is defined as the number of dissolved particles per kilogram of solvent. It can be measured by osmometer or calculated as a sum of the solutes present in the solution. The normal range of serum osmolality is 285–295 mOsm/kg. Sodium is the major determinant of serum osmolality. The formula given bellow is used for calculating serum osmolality:

serum osmolality=2×serum Na+serum K+glucose+urea(in mmol/l),

or formula used in the United States

$$\begin{array}{l} \text{serum osmolality}=2\times \text{serum Na}+\text{serum K}+\text{glucose}/\text{​}18+\text{BUN}/\text{​}2.8\\ (\text{Na and K are measured in mmol/l, glucose and BUN are measured in mg/dl}). \end{array}$$

Tonicity is the effective osmolality and measures the concentration of particles, which have the capacity to exert osmotic force across the membrane. Tonicity is determined only by solutes that cannot freely cross the semipermeable membrane. Thus, particles that move easily through the membrane (e.g., urea) are considered ineffective and do not contribute to tonicity. Tonicity may be calculated with the following equations (22):

serum tonicity=2×serum Na+serum K+glucose(in mmol/l),

$$\begin{array}{l} \text{serum tonicity}=2\times \text{serum Na}+\text{serum K}+\text{glucose/}18\\ (\text{Na and K are measured in mmol/l, glucose is measured in mg/dl}). \end{array}$$

**Figure 2:**
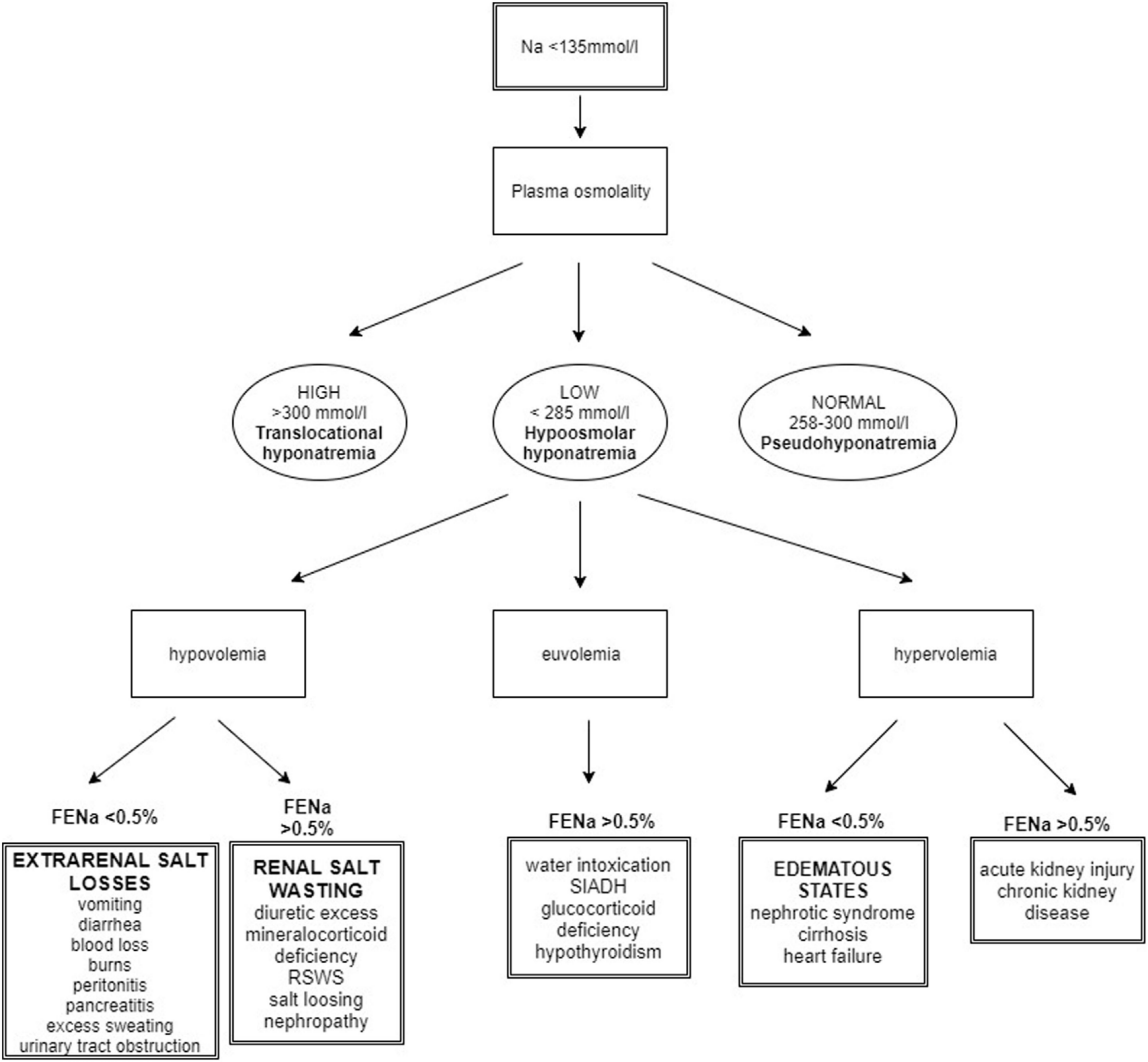
Algorithm for the approach to hyponatremic patient. Taking history is the initial step in hyponatremia evaluation. After the measurement of the plasma osmolality, the main task for the clinician is to assess the body fluid status. FENa calculation contributes to the final determination of diagnosis [modified from Ref. (18)].

Hyponatremia may be associated with serum isotonicity, hypertonicity, and hypotonicity. Differentiating these three tonic states is necessary for proper management. Assessment of the body fluid volume along with calculation of sodium fraction excretion (FENa) are further steps in the diagnostic evaluation of hyponatremia (18) (Figure 2).

## Isotonic Hyponatremia (Pseudohyponatremia)

Pseudohyponatremia is a condition where hyponatremia is due to the presence of hypertriglyceridemia, hypercholesterolemia, or hyperproteinemia. These conditions are associated with a reduction in the plasma water fraction and lower measured sodium concentration (23). Plasma water accounts for approximately 93% of total plasma volume. Assuming that the plasma sodium concentration is 140 mmol/l, the expected plasma water sodium concentration would be 150 mmol/l. Indirect ion-selective electrode (ISE) methods measure sodium concentration in total serum. The sample is diluted before measurement. Hyperproteinemia or hypertriglyceridemia increases the non-aqueous fraction of plasma; the serum then contains a lower volume of plasma water and less sodium. An assessment of sodium using the principle of direct ISE is independent of the content of solids in the sample. This method measures sodium activity in plasma of undiluted samples because indirect ISE falsely measures low sodium; direct ISE methods are used to avoid this problem (24).

## Hypertonic Hyponatremia

Excess of osmotically active non-electrolyte solutes in the ECF is characteristic for hypertonic hyponatremia. Serum osmolality is elevated to >295 mmol/l. Increased osmolality is associated with water gain and ECF dilution (25). Glucose is an osmotically active substance; hyperglycemia increases serum osmolality and results in water shifting out of the cells (26). Hypertonic hyponatremia can be caused also by manitol, sucrose, intravenous radiocontrast, and low molecular weight dextrans (24). Urea is an ineffective osmole and does not affect serum tonicity, thus uremia does not lead to hypertonic hyponatremia. Formulas for corrected Na calculation in hyperglycemic states are as follows (22):

$$\begin{array}{l} \text{corrected Na}=\text{measured serum Na}+(2.4\times \text{serum glucose}-5.5)\text{/}\\ 5.5(\text{in mmol/l}), \end{array}$$

$$\begin{array}{l} \text{corrected Na}=\text{measured serum Na}+(2.4\times \text{glucose}-100)/\\ 100(\text{Na is measured in mmol/l, glucose is measured in mg/dl}). \end{array}$$

## Hypotonic Hyponatremia

The most commonly encountered form of hyponatremia is hypotonic hyponatremia, which is associated with reduced serum osmolality (<275 mmol/l). It is further classified based on the patient’s extracellular volume status. Hyponatremia can occur with normovolemia, hypervolemia, or hypovolemia. Hypovolemia due to extrarenal losses most often due to gastroenteritis is the most common cause of hypovolemic hyponatremia. Patients typically have clinical symptoms of dehydration and are rehydrated with hypotonic fluids. Sodium fraction excretion (FENa) is low as the body preserves sodium. Hypovolemic hyponatremia with high FENa may be caused by diuretics, renal salt wasting syndrome (RSWS), or mineralocorticoid deficiency (27). Diuretics have a potent natriuretic effect. Hydrochlorothiazide causes hyponatremia by inhibition of a Na^+^Cl^−^ cotransporter on the luminal side of the distal tubule, reduction in diluting ability, and water excretion in a distal tubule (28). RSWS is defined as ECF depletion due to a sodium transport abnormality with or without high urinary sodium concentration. Adrenal and thyroid disease must be excluded as well as kidney function impairment (29). Although it was supposed to be associated only with intracranial disorders, it has been also linked to non-cerebral diseases. Amplification of the circulating NP may, according to some authors, lead to RSW. NP increase GFR and natriuresis, and they can also inhibit the action of aldosterone and reduce sympathetic outflow (30, 31). However, this theory was not supported by other studies, where low plasma levels of NP were found in patients with RSW (29, 32). It is presumed that initial defects in sodium reabsorption cause ECV depletion. Hypovolemia activates RAAS and ADH secretion to achieve equilibrium (33). Also inherited salt loosing nephropathies—Bartter and Gitelman syndrome and mineralocorticoid deficiency may cause hypovolemic hyponatremia. Euvolemic hyponatremia is characteristic for patients with a syndrome of inappropriate ADH secretion (SIADH), caused by inappropriately enhanced ADH secretion leading to volume expansion and subsequent increased natriuria due to NP and other factors (34). Higher FENa (>0.5%) is characteristic both for RSW and SIADH; it is challenging at times to distinguish between these two diseases (Table 3) (35). While RSW is associated with hypovolemia, patients with SIADH are euvolemic or mildly hypervolemic (34). Also, water intoxication may be the cause of euvolemic hyponatremia when the rate of water intake exceeds the rate of diuresis (36). Another potential cause of hyponatremia is hypothyroidism. The mechanisms are not completely clear, but both prerenal and renal causes are presumed (37). Patients with nephrotic syndrome, cirrhosis, and heart failure may develop hyponatremia with signs of clinical hypervo-lemia. These individuals are in reality hypovolemic, because the body senses only fluid in the vascular system, thus, intravascular dehydration enhances ADH secretion and water retention results in hyponatremia. Calculated FENa is usually <0.5%. On the other hand, individuals with acute kidney injury or chronic kidney disease can present with hypervolemic hyponatremia with high FENa (>0.5%) due to tubular dysfunction (18).

**Table 3:** Differentiation of SIAD from renal salt wasting syndrome (RSWS) [modified from Ref. (18)].

	SIAD	RSWS
Volume status	Euvolemia	Hypovolemia
Urine output	Low	High
Central venous pressure	Normal	Low
Urea and creatinine	Low/normal	High
Uric acid	Low	Low
Plasma osmolality	Decreased	Decreased
Urine Na concentration (mmol/l)	>20*	>20*
Plasma renin activity, aldosterone	Low	High

*Urine Na concentration may be <20mmol/l when sodium is restricted in the diet.

## Iatrogenic Hyponatremia

Iatrogenic hyponatremia is a common problem in hospitalized children. It may occur in children who are at risk of excessive ADH production. Potential stimuli are pain, nausea, stress, vomiting, and volume depletion. Administration of hypotonic fluid can result in severe hyponatremia with life-threatening neurologic complications. Fatal cases of hospital-acquired hyponatremia have been published in the literature (38). The use of isotonic maintenance fluids is the main preventive measure (39). Major clinical conditions associated with excessive ADH production are perioperative and postoperative states, pulmonary and central nervous system disorders, and oncologic diseases. Notably, children with psychiatric disorders are at significant risk for hyponatremia development. Some psychiatric drugs are associated with SIADH, and these children also suffer more often from habitual polydipsia. An excess of free water along with decreased water excretion may lead to symptomatic hyponatremia in these patients (40).

## Hyponatremia in Specific Clinical Situations

There are specific clinical situations associated with hyponatremia, which may also affect children and adolescents. 3,4-Methylenedioxymethamphetamine, also called ecstasy, is a synthetic amphetamine, a recreational drug popular among some adolescents and young adults. Ecstasy can lead to hyponatremia and serious neurologic complications due to inappropriate ADH release and high fluid intake. ADH secretion may in some cases be enhanced by other stimuli such as nausea and stress. Menstrual females are at a higher risk due to increased expression of receptors for ADH and a stronger serotoninergic response. Estrogens may also stimulate ADH secretion (41, 42). Also, endurance sport and exercise may cause hyponatremia. High fluid intake and sodium sweat loss along with inappropriate ADH secretion are responsible for hyponatremia development. Nausea, pain, and stress may also contribute to ADH release (43).

Feldman et al. published in 2005 a report of two infants with the symptomatology of chronic SIADH. Serum ADH levels were undetectable in both patients. Gain-of-function mutations of the X-linked V2 receptors that resulted in nephrogenic syndrome of inappropriate antidiuresis were identified in both patients. This disease represents a mirror image of nephrogenic diabetes insipidus (44).

## Hyponatremic Encephalopathy

Mild hyponatremia may be asymptomatic; however, the most feared complication is hyponatremic encephalopathy. Hypoosmolality of the ECF in hyponatremic states may cause brain edema, increased intracranial pressure, impaired cerebral blood flow, and sometimes herniation (45). Initial brain adaptation to hyponatremia consists of solute (Na, K, and the organic osmolytes—glycine, taurine, myoinositol, and creatine) and water expulsion to restore cell volume (46). Major risk factors for hyponatremic encephalopathy development include underlying central nervous system disease, female gender, hypoxia, and children aged <16 years. Children have less space for cerebral tissue expansion due to their larger brain to intracranial volume ratio compared with adults (47, 48). Menstruating women are at significant risk for the development of hyponatremia because estrogen stimulates ADH secretion. There is also a gender difference in renal endogenous ADH sensitivity caused by higher expression of renal V2 receptors for ADH (49, 50). Hypoxemia impairs brain cell volume regulation in response to hyponatremia. It is a major factor in the outcome of patients with hyponatremic encephalopathy (51). Symptoms of hyponatremic encephalopathy are usually nonspecific as patients can present with headache, nausea, vomiting, weakness, lethargy, and confusion. More severe symptoms include altered consciousness, seizure, coma, myocardial ischemia, and arrhythmias (47). Patients with hyponatremic encephalopathy may develop neurogenic pulmonary edema due to a centrally mediated increase in pulmonary vascular permeability, and pulmonary vasoconstriction caused by increased sympathetic neuronal activity and the release of catecholamines. Pulmonary injury further exacerbates cerebral edema in a vicious circle of worsening cerebral and pulmonary edema. This condition, first reported in postoperative and exercise-associated hyponatremia, was called Ayus–Arieff syndrome (52). Hyponatremic encephalopathy is a life-threatening medical emergency and requires immediate and effective treatment.

## Conclusion

Understanding pathophysiology is necessary for the correct evaluation and management of a hyponatremic patient. Appropriate or inappropriate increased secretion of ADH with water retention is the most common cause of hyponatremia. The main task for the physician is to differentiate between conditions with an abundance of free water and states with salt loss because the treatment differs depending on etiology. Hyponatremic encephalopathy is a severe condition that may be difficult to recognize. Thus, assessment of natremia is warranted in patients with nonspecific symptoms.

## Author Contributions

The author confirms being the sole contributor of this work and approved it for publication.

## Conflict of Interest Statement

The author declares that the research was conducted in the absence of any commercial or financial relationships that could be construed as a potential conflict of interest.
